# Supplementary material for: Electromyography of Extrinsic and Intrinsic Ear Muscles in Healthy Probands and Patients with Unilateral Postparalytic Facial Synkinesis
Source: Diagnostics (Basel). 2022 Jan 5;12(1):121. doi: 10.3390/diagnostics12010121 (PMC8775077; doi:10.3390/diagnostics12010121)
Supplement: Supplementary file 1 [file diagnostics-12-00121-s001.zip › diagnostics-1517827-supplementary.pdf]

Supplementary Materials

Supplementary Table S1: Needle single-channel EMG activity* in the different ear muscles during mimic tasks on right and left side in one healthy probands.

Muscle	Smiling		Pursing Lips		Nose Wrinkling		Frowning		Drawing Eyes Brows		Ipsilateral Gaze		Contralateral Gaze		Ear Wiggling	
	Right	Left	Right	Left	Right	Left	Right	Left	Right	Left	Right	Left	Right	Left	Right	Left
Auricularis anterior	3	0	0	0	0	0	1	1	0	0	0	0	0	0	2	3
Auricularis superior	3	3	0	0	2	0	2	3	0	0	0	0	0	0	3	3
Auricularis posterior	2	0	1	0	0	0	3	3	0	0	1	0	0	0	3	3
Tragicus	2	2	0	3	0	0	0	0	3	0	0	0	0	0	1	2
Antitragicus	3	2	0	0	0	0	0	2	2	0	0	0	0	0	2	3
Helicis major	3	3	0	0	0	0	0	0	0	3	0	0	0	0	2	2
Helicis minor	2	2	0	0	0	0	0	0	0	0	0	0	0	0	2	2
Transversus auriculae	X	0	X	2	X	X	X	0	X	0	X	3	X	3	X	2
Obliquus auriculae	2	X	0	X	0	X	0	X	0	X	0	X	0	X	1	X

*EMG classification: 0 = no increase compared to baseline activity in resting state, 1= slight increase, 2 = moderate increase, 3 = strong increase; X = not analyzable. No means ± standard deviation are reported as only one proband was examined.

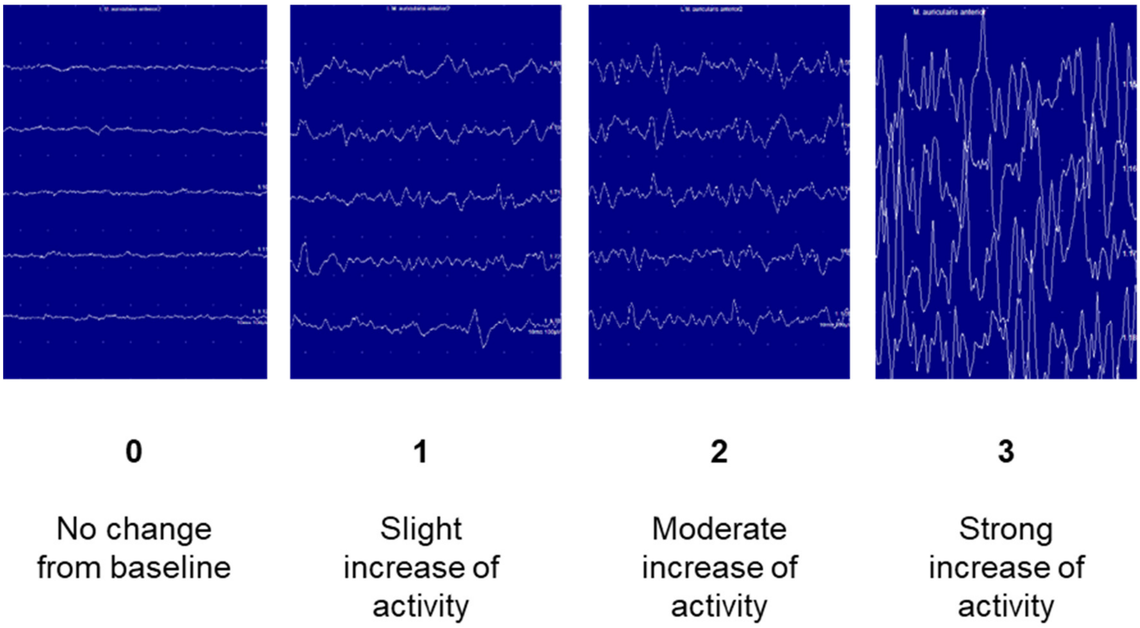

Supplementary Figure S1. Classification system for the EMG activity during the mimic tasks.

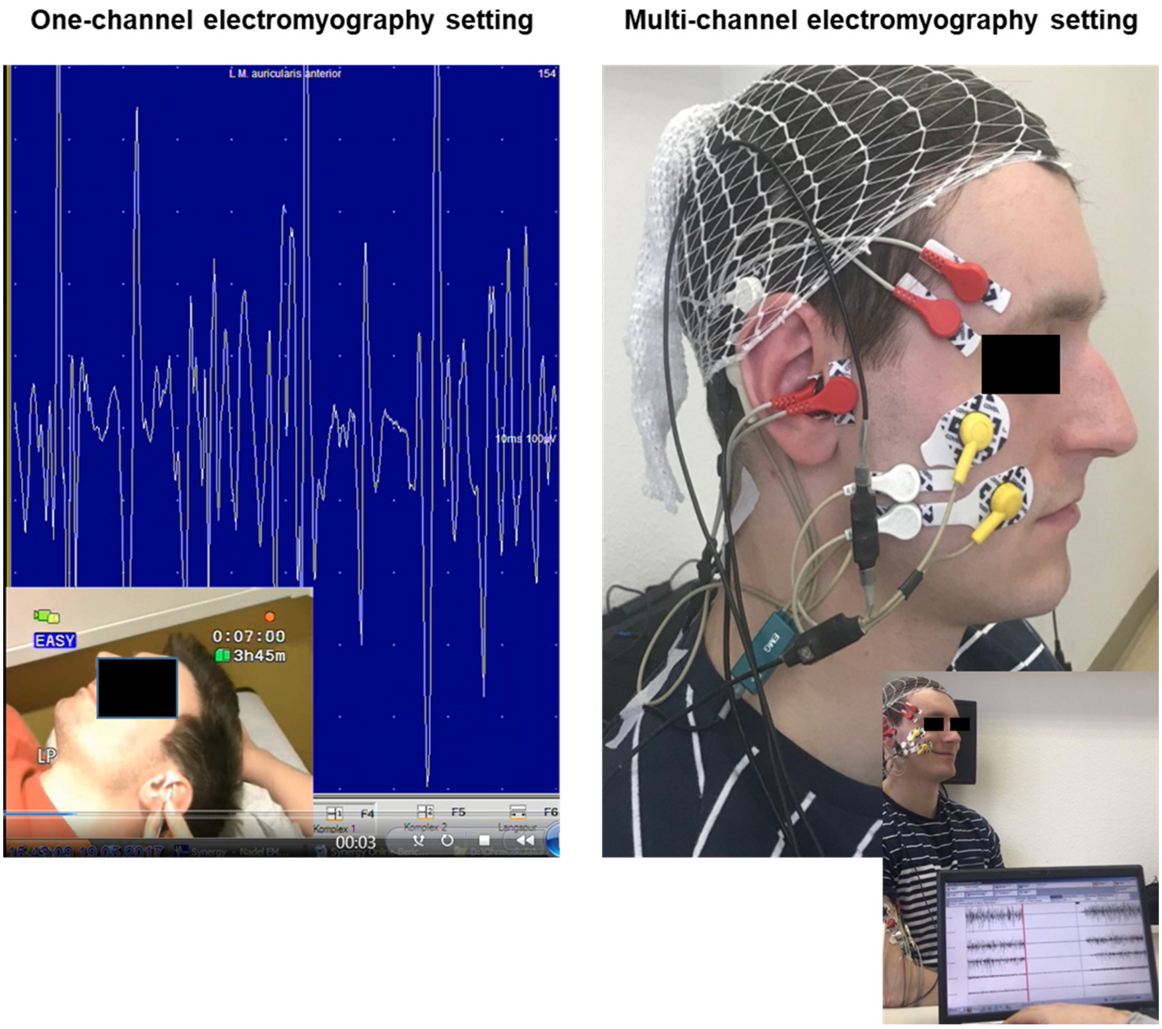

Supplementary Figure S2. Example for the setting of the single-channel EMG recording with a patient in supine position on the left side and for multi-channel surface EMG recordings on the right side.

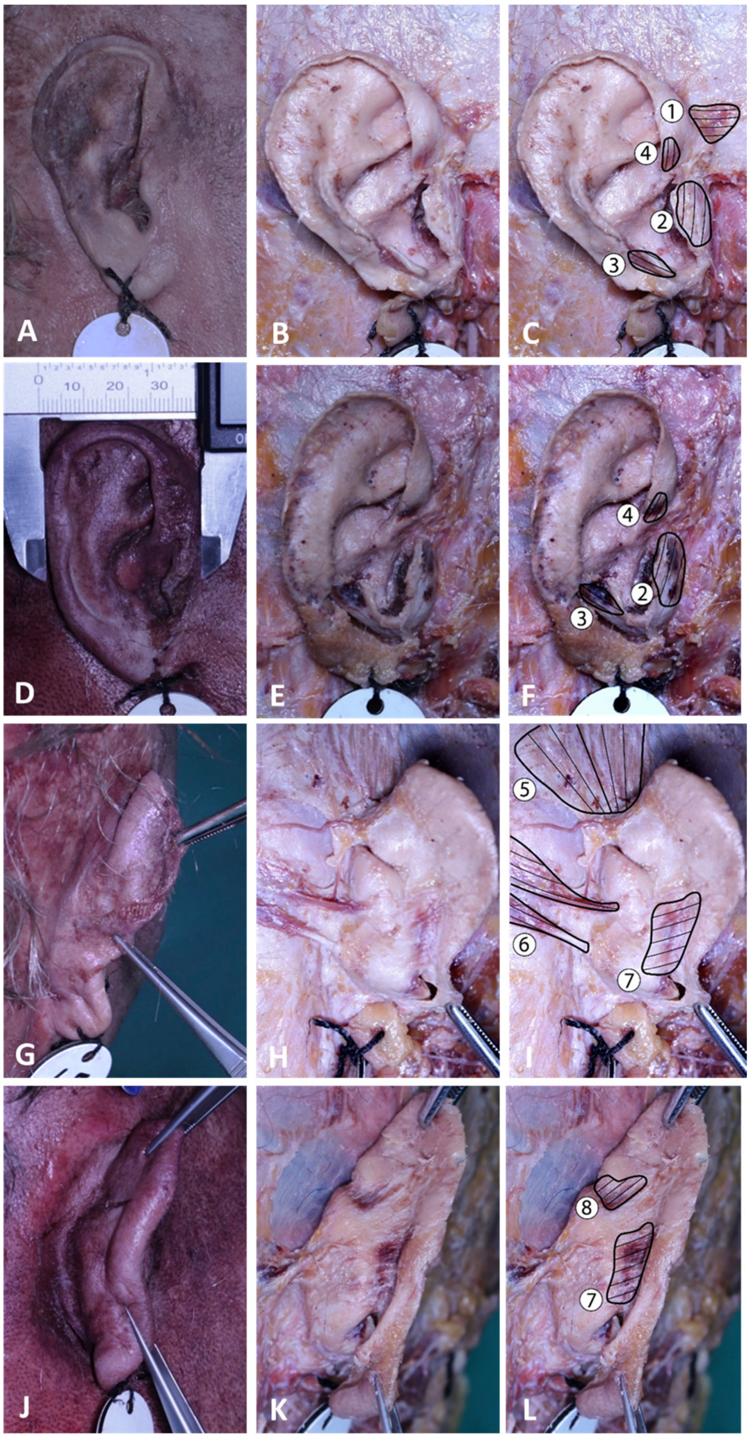

Supplementary Figure S3. Cadaver preparations of the ear muscles. (**A**–**C**), (**G**–**I**): dissection no. 1; (**D**–**F**), (**J**–**L**): dissection no. 2. (**A**–**F**): anterior side of both dissections; (**G**–**L**): posterior side of both dissections. A/D/G/J: before dissection; B/E/H/K: after dissection; C/F/I/L: dissected muscles delineated. 1 = M. auricularis anterior, 2 = M. tragicus, 3 = M. antitragicus, 4 = M. helicis major, 5 = M. auricularis superior, 6 = M. auricularis posterior, 7 = M. transversus auriculae, 8 = M. obliquus auriculae.

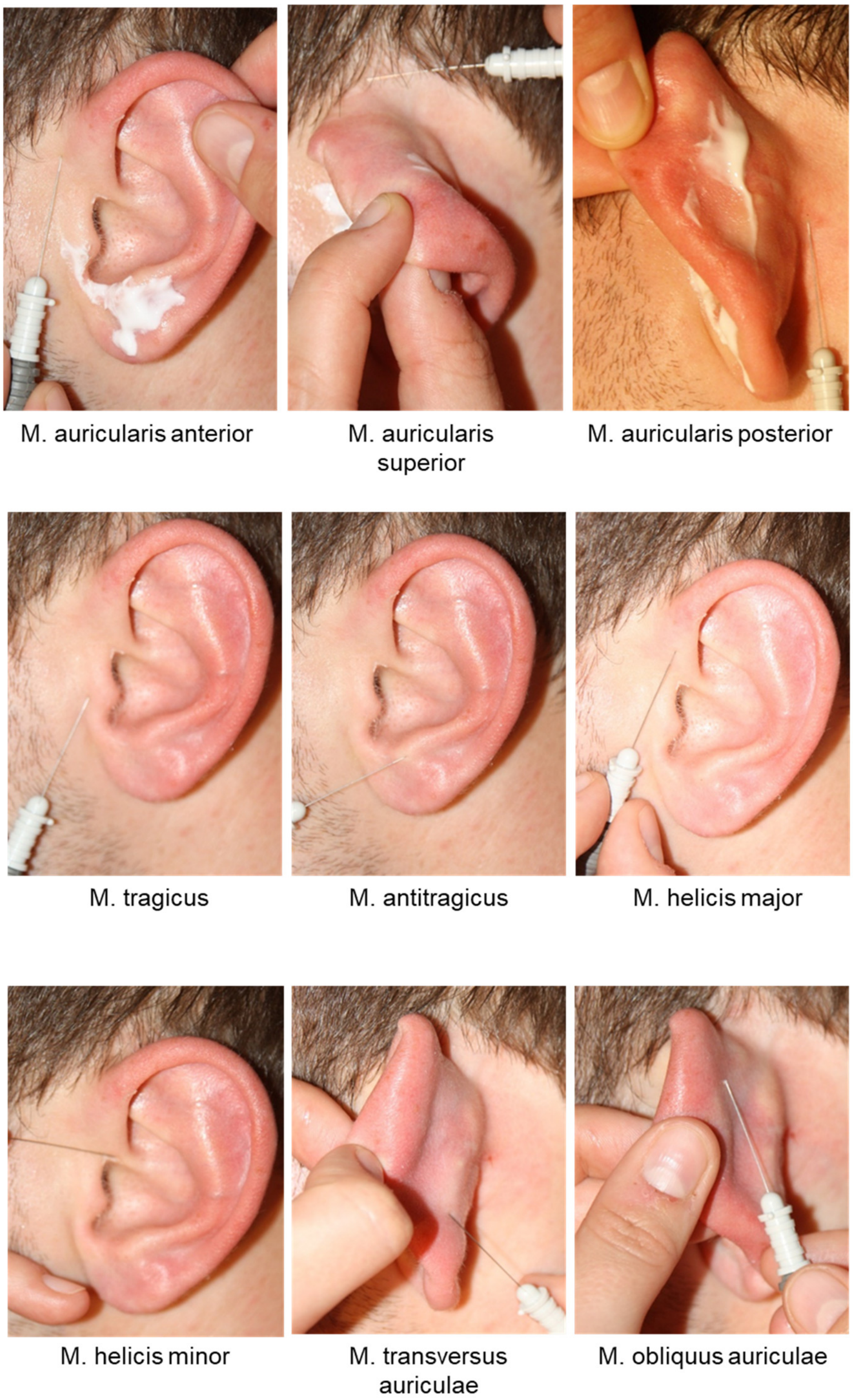

Supplementary Figure S4. Hot spots for needle EMG for each individual ear muscle shown on the left ear.
